# Long-term outcome of the Sauvé-Kapandji procedure in patients with post-traumatic distal radioulnar joint disorders

**DOI:** 10.1177/17531934241242678

**Published:** 2024-03-27

**Authors:** Gijs P. Debeij, Pascal F. W. Hannemann, Jan A. Ten Bosch

**Affiliations:** Department of Surgery, Division of Trauma Surgery, Maastricht University Medical Center+, Maastricht, The Netherlands

**Keywords:** Distal radioulnar joint, Sauvé-Kapandji, patient-reported outcomes, osteotomy, pseudoarthrosis

## Abstract

The aims of this study were to retrospectively assess the occurrence of complications or need for secondary wrist procedures after the Sauvé-Kapandji procedure, and to prospectively assess patient-reported outcomes at long-term follow-up. All patients treated with the Sauvé-Kapandji procedure in our tertiary referral hospital between January 2008 and September 2021 were identified and contacted to complete the Quick Disabilities of the Arm, Shoulder, and Hand and the Patient-Rated Wrist/Hand Evaluation outcome measures. In total, 30 patients, with a median follow-up of 82 months, were included in this study. Complications occurred in 6 of 30 patients, which resulted in six secondary wrist procedures. Mean Quick Disabilities of the Arm, Shoulder, and Hand and Patient-Rated Wrist/Hand Evaluation scores were 30.1 and 33.6, respectively. We conclude that in respect of long-term outcomes, the Sauvé-Kapandji procedure can still be deemed to be a useful procedure, especially in patients with few other reconstructive options.

**Level of evidence:** IV

## Introduction

Distal radioulnar joint (DRUJ) disorders can result in chronic wrist pain, weakness of grip strength and limited range of motion (ROM). To treat these problems, an arthrodesis of the DRUJ can be combined with an ulnar pseudoarthrosis just proximal to the arthrodesis by ulnar resection osteotomy. This method is known as the Sauvé-Kapandji procedure.

Over the last decade, there have been several reports about the Sauvé-Kapandji procedure. These studies provide mixed evidence in terms of outcome. Six studies report good mid- to long-term outcomes with adequate postoperative pain relief and an associated improved ROM (Giberson-Chen et al., 2020; Ikeda et al., 2018; Minami et al., 2018; Monreal and Faedo, 2016; Munaretto et al., 2020; Papp et al., 2013). Other studies have reported the incidence of long-term complications, including an unstable proximal stump, radioulnar convergence, symptoms of irritability of the dorsal sensory branch of the ulnar nerve, heterotopic ossification, hardware irritation and nonunion of the arthrodesis site, leading to reoperations (Minami et al., 1995; Nakamura et al., 1992; Reissner et al., 2021; Verhiel et al., 2021). However, it has been stated that these problems are relatively rare and can be reduced by careful surgical techniques (Giberson-Chen et al., 2020; Lluch, 2013).

Current studies feature either a long follow-up period with a relatively small study group or a larger study group with a relatively brief follow-up. Since consensus regarding the correct indications for treatment is lacking and various complications have been reported, we have conducted a retrospective study on the long-term outcome of the Sauvé-Kapandji procedure for the treatment of patients with disorders of the DRUJ after upper extremity injuries.

## Methods

Institutional review board approval was obtained to conduct a retrospective review of patients who had been treated with the Sauvé-Kapandji procedure. The patients were identified from the upper extremity trauma database, covering the period between January 2008 and September 2021. Our university hospital is a tertiary referral centre for hand and wrist pathology, providing specialized treatment for upper extremity injuries. In the present study, the Sauvé-Kapandji procedures, as described by Slater (2008), were carried out by three level 4 (Tang and Giddins, 2016) hand surgeons (PH, JTB and another [not an author]).

**Figure 1.**
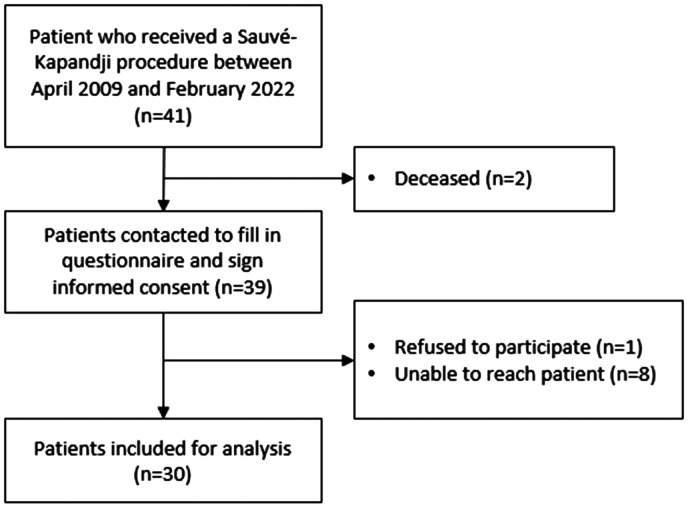
Inclusion flow chart.

To ensure a comprehensive analysis, all patients who underwent the Sauvé-Kapandji procedure during the specified inclusion period (*n* = 41) were screened and approached for potential participation in this study. In total, 11 patients were subsequently excluded from the final analyses (Figure 1).

Information was extracted retrospectively from the hospital medical charts, including baseline characteristics, operation history, operative details, preoperative function, peri- and postoperative complications, and any subsequent secondary procedures. Baseline characteristics were defined as the data immediately preceding the Sauvé-Kapandji procedure.

Long-term patient-reported outcomes were recorded using the Quick Disabilities of the Arm, Shoulder, and Hand (QuickDASH) (Beaton et al., 2005) and the Patient-Rated Wrist/Hand Evaluation (PRWHE) (MacDermid, 2019) questionnaires. These questionnaires, accompanied by informed consent forms to be signed, were sent to all eligible patients via mail. Before this, patients were contacted by phone to obtain permission for participation in the study.

### Data presentation

The distribution of data was checked using the Kolmogorov–Smirnov test. Depending on the results, continuous values are presented as mean (SD; range) or median (interquartile range [IQR]; range). Categorical data are presented as total numbers (*n*).

## Results

A total of 30 patients who underwent the Sauvé-Kapandji procedure were included in this study. Their baseline characteristics are shown in Table 1.

**Table 1.** Baseline characteristics.

Variable	Value
Sex (male/female) (*n* = 30)	16/14
Age (*n = *30)	
Age at procedure (years)	50 (18; 20–85)
Current age (years)	57 (18; 22–90)
Height (cm) (*n* = 28)	174 (12; 155–198)
Weight (kg) (*n* = 28)	77.3 (15.2; 55–108)
BMI (*n* = 28)	25.4 (3.5; 18.4–31.9)
Smoking status (non-smoker/former/current) (*n* = 28)	17/5/6
Pack years (*n* = 10)	13.3 (8.1; 3–30)
Alcohol status (no/yes) (*n* = 28)	15/13
Units of alcohol per week (*n* = 13)	3 (2 to 14; 1–25)

Data expressed as *n*, mean (SD; range) or median (IQR; range). *n* is the number of participants with relevant data.

BMI: body mass index (calculated as kg/m^2^).

**Figure 2.**
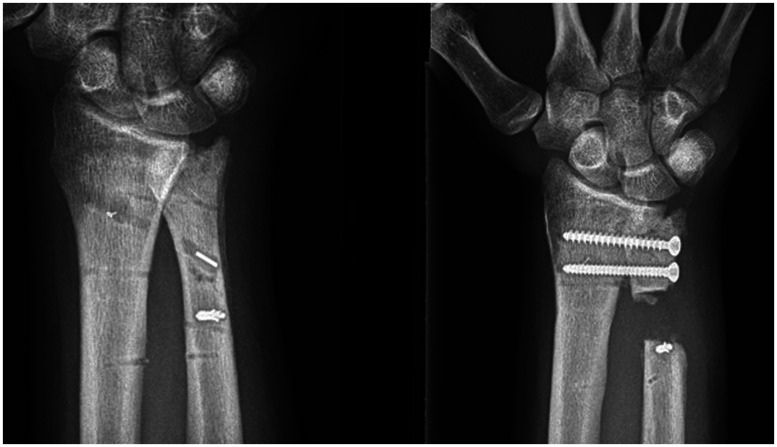
(Left) Preoperative and (right) postoperative radiographs of a patient in this study. The postoperative image shows a clear view of the screws and pseudoarthrosis.

The main indications for a Sauvé-Kapandji procedure are given in Table 2. Most patients (19/30) had undergone operative treatment for a fracture of the distal radius or the ulna, or both, before the Sauvé-Kapandji procedure (Figure 2).

**Table 2.** Injury and procedure characteristics.

Variable (*n* = 29)	Value
Dominant hand (right/left)	25/4
Affected hand (right/left/both)	13/15/1
Dominant hand = affected hand	17
Indication for SK	
Arthrosis after DR fracture	11
Malunion after DR fracture	12
Instability of DRUJ	3
Rotational impairment	3
Status after debridement due to septic arthritis	1
Concurrent procedures	17
Operative fracture treatment of distal radius and/or ulna before SK procedure	18
Occurrence of preoperative pain (*n* = 28)	24
Occurrence of preoperative instability (*n* = 27)	11
Preoperative pronation (°) (*n* = 22)	73 (34 to 88; 0–90)
Preoperative supination (°) (*n* = 27)	30 (0 to 58; 0–90)
Occurrence of perioperative complications	0
Occurrence of postoperative complications	6
Reoperation needed after SK procedure	6

Data expressed as *n* or median (IQR; range). *n* is the number of participants with relevant data.

DR: distal radius; DRUJ: distal radioulnar joint; SK: Sauvé-Kapandji.

Of the 30 included patients, 17 underwent a concurrent procedure with the Sauvé-Kapandji procedure. These concurrent procedures were removal of osteosynthesis material (*n* = 8), total wrist fusion (*n* = 4), partial radiocarpal arthrodesis (*n* = 2), correction osteotomy (*n* = 2), and debridement and necrotomy (*n* = 1).

Follow-up data for one patient were not available in the hospital file. Therefore, this individual was excluded from the complications section. Short-term perioperative complications (<6 weeks) did not occur. During follow-up, postoperative complications occurred in 6 of the 29 patients (Table 2). Four patients developed proximal ulnar stump instability, one patient had an osteomyelitis of the ulnar stump (this was not the patient with concurrent debridement and necrotomy) and one patient developed an ulnar positive variance with ulnocarpal impaction after 5 years. All these postoperative complications resulted in a subsequent reoperation. These reoperations included four semi-constrained DRUJ prostheses (Aptis Distal Radioulnar Joint Prosthesis; Aptis Medical, Louisville, KY, USA), one DAIR (debridement, antibiotics and implant retainment) procedure and one ulnar shortening osteotomy.

The patient-reported outcome measures (questionnaires) were completed by 28 of the 30 participants. These measures were assessed over a median follow-up period of 83 months (IQR 75 to 127; range 18–158). There was a mean PRWHE score of 33.6 (SD 21.0; range 2–70) and a mean QuickDASH score of 30.1 (SD 21.2; range 0–72.7). Concerning the PRWHE questionnaire, it was observed that pain-related questions received a mean score of 3.9 (SD 1.2) out of 10, while functional-related questions garnered a score of 2.9 (SD 0.7) out of 10. Similarly, the QuickDASH questionnaire revealed that pain-related questions received a mean score of 2.0 (SD 0.5) out of 5, while functional-related questions obtained a score of 2.3 (SD 0.5) out of 5.

## Discussion

The Sauvé-Kapandji procedure in the present study demonstrates postoperative complications in 6 of 29 patients, i.e. a long-term success rate of 79%. Combined with mean patient-perceived outcomes of 33.6 (PRWHE) and 30.1 (QuickDASH), this procedure demonstrates a very acceptable long-term outcome for patients with severe DRUJ pathology. The patient-reported outcomes in our study are in line with previous studies. Reissner et al. (2021) reported a mean PRWHE score of 30 out of 100. Giberson-Chen et al. (2020), Roulet et al. (2021) and Ikeda et al. (2018) reported QuickDASH scores after Sauvé-Kapandji procedures of 28, 26.5 and 22, respectively, which are slightly lower than the QuickDASH score of 30.1 in the present study.

A remarkable advantage of the Sauvé-Kapandji procedure is its potential for conversion to alternative procedures. This option can be used if the Sauvé-Kapandji procedure fails to adequately reduce the symptoms of DRUJ pathology or when specific complications occur. Conversion to a semi-constrained DRUJ prostheses, such as the Aptis Distal Radioulnar Joint Prosthesis, seems to be a solution in these situations with high patient satisfaction scores and low DASH and PRWE scores (Kachooei et al., 2014). However, they are not free from complications. Extensor carpi ulnaris tenosynovitis, heterotopic ossification, screw loosening, radial plate loosening, tendon irritation, carpal tunnel syndrome, lateral elbow pain, stem loosening and heterotopic ossification, and superficial radial nerve irritation have been reported (Axelsson and Sollerman, 2013; Kachooei et al., 2014; Lambrecht et al., 2022; Reissner et al., 2016; Scheker and Martineau, 2013). A higher mean DASH score of 40 and a PRWE score of 49 have also been reported (Lambrecht et al., 2022).

The Sauvé-Kapandji procedure can also be converted into a Darrach procedure, which has similar QuickDASH outcomes, but which has more mechanical symptoms owing to ulnar stump instability (Lamont et al., 2022). A primary Darrach procedure involves the resection of the distal part of the ulna, leading to a shorter ulnar stump, which can result in relative instability at the distal radioulnar joint. In contrast, the Sauvé Kapandji procedure preserves the distal ulna, allowing for better stability and a more physiological load transmission across the DRUJ, thus reducing the likelihood of mechanical symptoms in comparison with the Darrach procedure.

It should be emphasized that although converting the Sauvé-Kapandji to an alternative procedure is feasible, the reverse is not possible.

The limitations of this study primarily stem from its retrospective design. The absence of reliable pre- and postoperative physical examinations, such as ROM, grip strength or assessment of ulnar instability, limits the full assessment of functional outcomes and potential complications associated with the procedure. Objective measures are valuable, but the patient’s subjective outcome is more important. Improvements in quality of life hinge on addressing their personal experience, which can differ from objective data.

Another limitation that should be acknowledged is the potential for selection bias. As this study was retrospective in nature, the decision to carry out the Sauvé-Kapandji procedure to a patient was reached through a process of considering all available options. The decision to select certain patients for this procedure could have introduced bias, affecting the generalizability of the results. Information bias may have been present because of the use of patient records as the primary data source. It is worth noting that not all the information was available for every patient, potentially leading to biases in the results; however, missing information was relatively low in this study and unlikely to have a significant effect on the results.

A significant proportion of the patients underwent concurrent procedures, which might have confounded the results, making it difficult to attribute the observed outcomes solely to the Sauvé-Kapandji procedure. Nevertheless, these concurrent procedures are not expected to exert much effect on the wrist’s functionality, thereby limiting their impact on the overall functional outcomes.

In conclusion, despite the limitations inherent in this retrospective study, the Sauvé-Kapandji procedure had an acceptable long-term success rate with favourable patient-reported outcomes. The present study underscores the continued relevance of the Sauvé-Kapandji procedure in the treatment of DRUJ disorders. With its proven track record and established success, the Sauvé-Kapandji procedure should not be overlooked or dismissed as obsolete.
